# Contribution of life course cardiovascular risk factors to racial disparities in dementia incidence

**DOI:** 10.3389/frdem.2023.1215904

**Published:** 2023-06-29

**Authors:** Erin L. Ferguson, Eric Vittinghoff, Adina Zeki Al Hazzouri, Norrina Allen, Annette Fitzpatrick, Kristine Yaffe

**Affiliations:** ^1^Department of Epidemiology and Biostatistics, University of California, San Francisco, San Francisco, CA, United States; ^2^Department of Epidemiology, Columbia University Mailman School of Public Health, New York, NY, United States; ^3^Department of Internal Medicine, Northwestern University, Chicago, IL, United States; ^4^Departments of Family Medicine, Epidemiology, and Global Health, School of Public Health, University of Washington, Seattle, Washington, DC, United States; ^5^Department of Psychiatry and Behavioral Sciences, Weill Institute for Neurosciences, University of California, San Francisco, San Francisco, CA, United States; ^6^Department of Neurology, Weill Institute for Neurosciences, University of California, San Francisco, San Francisco, CA, United States

**Keywords:** health disparity populations, risk factors, mediation analysis, imputation, life course, heart disease risk factors

## Abstract

**Background:**

Racial disparities in dementia outcomes persist in the United States. Targeting modifiable risk factors, including cardiovascular risk factors (CVRFs), is a conceivable way to reduce health disparities. Life course CVRFs are often higher in non-White adults and are associated with risk of dementia, but it is unknown whether they contribute to racial disparities in dementia and cognition.

**Methods:**

Using a pooled cohort of 4,159 White and 939 Black participants aged 65–95 years, we conducted a mediation analysis to estimate the proportional effect of race on dementia that is explained by four CVRFs imputed over the life course (20–49, 50–69, and 70–89 years of age): body mass index, fasting glucose, systolic blood pressure, and low-density lipoprotein cholesterol.

**Results:**

Compared to White participants, Black participants had greater risk of dementia (adjusted OR = 1.37; 95% CI: 1.17–1.60). BMI and fasting glucose over the life course were significant mediators of the effect of race on dementia risk, mediating 39.1% (95% CI: 10.5–67.8%) and 8.2% (95% CI: 0.1–16.2%) of the effect, adjusted for sex and age. All four CVRFs together were also significant mediators of the effect of race on scores on global cognition and processing speed, accounting for approximately 11% of the effect.

**Conclusions:**

We found that CVRFs across the life course partially explain disparities in dementia risk and cognition in late-life. Improved prevention and treatment of CVRFs across the life course may be important to reduce health disparities for dementia.

## 1. Introduction

In the US, Black adults have been shown to have consistently higher incidence and prevalence of dementia compared to non-Hispanic White adults (Mayeda et al., 2016; Steenland et al., 2016; Matthews et al., 2019). Previous literature suggests that this disparity can be partially explained by social determinants of health (SDOH), such as socioeconomic status (Yaffe et al., 2013; Samuel et al., 2020), education (Farina et al., 2020), and inequities in access to quality and timely healthcare (Saadi et al., 2017; Kawas et al., 2021; Murchison et al., 2021). These factors are all largely downstream effects of structural and institutionalized racism that interact to contribute to inequities in dementia incidence (Caunca et al., 2020).

These disparities may also stem from differences in cumulative cardiovascular risk factors (CVRFs). CVRFs, such as obesity and hypertension, are known risk factors for cognitive decline and dementia (Whitmer et al., 2005; Alonso et al., 2009; Qiu and Fratiglioni, 2015; Livingston et al., 2020; Yaffe et al., 2021; Zeki Al Hazzouri et al., 2021). CVRFs are very common (Heart Disease and Stroke Statistics–2019 Update: A Report From the American Heart Association | Circulation, n.d.) and can be treated with lifestyle changes (Pan et al., 2018; Rippe, 2018; Lavie et al., 2019) or medical treatment (Field et al., 1995; Jackson et al., 2005), which make them critical targets for intervention. Because the prevalence of CVRFs are higher in Black and Hispanic adults compared to White adults (Lewey and Choudhry, 2014; Howard et al., 2017; Bell et al., 2018), they may also contribute to observed racial disparities in dementia incidence (Chen and Zissimopoulos, 2018). To fully capture the contributions of CVRFs to racial disparities, it is important to consider exposure to CVRFs over early, mid, and late-life. However, most studies of CVRFs and dementia incidence have only examined mid or late-life exposures because of the lack of cohort studies spanning the entire adulthood period.

Using data from a racially/ethnically diverse pooled cohort of Black and White individuals based on four established studies spanning over 7 decades, we investigate whether CVRFs over the adult life course (early adult, mid, and late-life) contribute to racial disparities in dementia risk and cognition. Since prevalence of CVRFs differ observationally by Black or White race (Lewey and Choudhry, 2014; Howard et al., 2017; Bell et al., 2018), we hypothesize that racial disparities in dementia incidence are partially mediated by CVRFs.

## 2. Methods

### 2.1. Parent pooled cohort

Data were pooled from 4 prospective cohorts: the Coronary Artery Risk Development in Young Adults study (CARDIA, an early adult to midlife cohort), the Multi-Ethnic Study of Atherosclerosis (MESA, midlife cohort), the Cardiovascular Health study (CHS, late-life cohort), and the Health, Aging, and Body Composition study (Health ABC, late-life cohort). Participants in each of the four cohorts self-reported their racial identity at enrollment. Additionally, each cohort collected self-reported information on education attainment (less than high school, completion of high school, some college, college graduate, and graduate/professional degree). The pooled cohort included 15,001 adult individuals aged 18–95 at enrollment, with at least 2 repeated measures of each of the measured CVRFs. Details of this pooled cohort are published elsewhere (Zeki Al Hazzouri et al., 2019).

### 2.2. Life course CVD risk factors

Data from the four prospective cohorts, that together spanned the adult life course: early and midlife data from MESA and CARDIA (*n* = 9,903), and late-life data from CHS and Health ABC (*n* = 5,098) were used to model life course CVRF trajectories encompassing early, mid, and late-life. Our late-life cohort participants, *n* = 5,098, had repeated measures of cognition and dementia outcome data and were the focus of outcome results. We focused on modeling the following four CVRFs: body mass index (BMI), fasting glucose (FG), systolic blood pressure (SBP), and low-density lipoprotein cholesterol (LDL). Using data from all four cohorts, we fit person-specific linear mixed models to estimate flexible trajectories of CVRFs over the life course. This imputation model was adjusted for cohort, demographic information (race, sex, splines in age and birth year, and interactions between age, sex, and race) and time-dependent clinical information (diabetes, hypertension, lipid-lowering and anti- hypertensive medication use, and smoking status). Imputations on these covariates were run prior to imputing CVRF trajectories. The imputation models additionally contained random intercepts and random age splines. As the following analysis depends on the accuracy of these imputation procedures, we conducted a simulation study treating the modeled trajectories of CVRFs as the true values and then used these values to model new trajectories. This analysis found that the imputation errors sometimes induced attenuation bias in regression coefficients, biasing toward the null. Further details about the imputation of CVRFs, its validation, and this simulation study are previously published (Zeki Al Hazzouri et al., 2019).

From these imputations, we estimated CVRF cumulative time-weighted averages (TWAs) for each participant in CHS and Health ABC at the three age ranges: early adulthood (ages 20–49), midlife (ages 50–69), and late-life (ages 70–89) (Table 1). For each age range, average cumulative period-specific CVRF TWAs were categorized into three levels based on standard clinical cutoffs: BMI (<25 kg/m^2^, 25–30 kg/m^2^, >30 kg/m^2^), SBP (<120 mmHg, 120–140 mmHg, >140 mmHg), FG (<100 mg/dL, 100–125 mg/dL, >125 mg/dL), and LDL (<100 mg/dL, 100–130 mg/dL, >130 mg/dL).

**Table 1:** Imputed life course Cardiovascular Risk Factor (CVRF) time-weighted averages (TWAs) by race.

	**Standard clinical cutoffs values are** ***N*** **(%)**					
	**White (*****n*** = **4.159)**			**Black (*****n*** = **939)**		
	**Highest category**	**Middle category**	**Lowest category**	**Highest category**	**Middle category**	**Lowest category**
**BMI (kg/m** ^2^ **)**						
Early adulthood	90 (2.2)	770 (18.5)	3,299 (79.3)	157 (16.7)	393 (41.9)	389 (41.4)
Midlife	623 (15)	1,821 (43.8)	1,715 (41.2)	395 (42.1)	370 (39.4)	174 (18.5)
Late-life	455 (10.9)	1,388 (33.4)	2,316 (55.7)	316 (33.7)	353 (37.6)	270 (28.8)
**SBP (mmHg)**						
Early adulthood	19 (0.5)	2,297 (55.2)	1,843 (44.3)	12 (1.3)	725 (77.2)	202 (21.5)
Midlife	822 (19.8)	2,462 (59.2)	875 (21.0)	362 (38.6)	492 (52.4)	85 (9.1)
Late-life	897 (21.6)	1,673 (40.2)	1,589 (38.2)	361 (38.4)	430 (45.8)	148 (15.8)
**LDL (mg/dL)**						
Early adulthood	1,113 (26.8)	2,856 (68.7)	190 (4.6)	214 (22.8)	645 (68.7)	80 (8.5)
Midlife	1,868 (44.9)	1,947 (46.8)	344 (8.3)	348 (37.1)	459 (48.9)	132 (14.1)
Late-life	1,171 (28.2)	1,425 (34.3)	1,563 (37.6)	301 (32.1)	394 (42.0)	244 (26.0)
**FG (mg/dL)**						
Early adulthood	6 (0.1)	190 (4.6)	3,963 (95.3)	3 (0.3)	47 (5)	889 (94.7)
Midlife	297 (7.1)	1,034 (24.9)	2,828 (68.0)	123 (13.1)	199 (21.2)	617 (65.7)
Late-life	321 (7.7)	935 (22.5)	2,903 (69.8)	138 (14.7)	217 (23.1)	584 (62.2)

CVRF cumulative time-weighted averages (TWAs) for early adulthood (age 20–49), midlife (age 50–69), and late-life (age 70–89) were categorized into three groups based on the following clinical cutoffs: Body mass index (<25 kg/m^2^, 25–30 kg/m^2^, >30 kg/m^2^), systolic blood pressure (<120 mmHg, 120–140 mmHg, >140 mmHg), low-density lipoprotein cholesterol (<100 mg/dL, 100–130 mg/dL, > 130 mg/dL), and fasting glucose (<100 mg/dL, 100–125 mg/dL, >125 mg/dL).

### 2.3. Dementia and cognitive outcomes

Dementia diagnoses were ascertained in the older two cohorts (CHS and Health ABC) by each study protocol. In CHS, participants at risk of dementia were administered a battery of neuropsychological tests, and those who failed the memory test or two other cognitive domains were evaluated by a neurologist. Dementia diagnoses were determined by a clinical adjudication committee (Lopez et al., 2003). In Health ABC, dementia was defined using previously published classifications (Hong et al., 2013; Brenowitz et al., 2021). Over 15 years of follow-up, participants with documented use of dementia medication, a hospitalization with dementia as primary or secondary diagnosis, or clinically meaningful global cognitive decline, defined as a >1.5 SD change, were classified as having dementia (Zeki Al Hazzouri et al., 2021).

Cognition was measured using the Digit Symbol Substitution Test (DSST), a test of processing speed, and the Modified Mini-Mental State Examination (3MS), a test of global cognition, annually in the CHS cohort and every 1–2 years in the Health ABC cohort.

### 2.4. Statistical analysis

We used Baron and Kenny decomposition methods to quantify mediation by CVRFs (Baron and Kenny, 1986), which is used frequently in the CVRF and cognition literature (Cui et al., 2020; Hu et al., 2022; Wang et al., 2022). First, base pooled logistic regression models adjusting for age and sex were fit for the association between race and dementia risk. Next, augmented pooled logistic regression models were fit for dementia from race, age, and sex, but additionally adjusting for each period-specific CVRF TWAs. In other words, the augmented models are the base models with adjustment for CVRFs in early adulthood, midlife, and late-life together. Mediation by cumulative CVRFs was summarized by the change in the coefficient for race, as adjusting for the mediator should reduce the total effect of race in the base model by blocking the mediating pathway through CVRFs (Baron and Kenny, 1986; Fairchild and McDaniel, 2017). We report the relative change in the coefficient for race as the proportion of the effect mediated by CVRFs, adjusted for age and sex. In subsequent mediation models, we additionally adjusted for education attainment in both base and augmented models (less than high school, completion of high school, some college, college graduate, and graduate/professional degree).

We also conducted mediation analyses using cognition (DSST and 3MS) as our secondary outcome. For these analyses, we used the same Baron and Kenny decomposition approach. We first fit linear regression models for cognition from race, adjusting for age and sex. Then, we fit augmented linear regressions, which were the same as the base model with adjustment for each period-specific CVRF TWAs. Mediation was again summarized by the change in coefficient for race between the base and augmented models. All analyses were conducted using Stata Version 16.1 (Stata LLP, College Station, TX).

## 3. Results

Our primary analysis for late-life dementia included 4,159 White and 939 Black participants from CHS and Health ABC. At baseline, Black participants were older (73.1 years vs. 72.5 years), more likely to be female (59.9 vs. 54.5%), and less likely to have completed college-level education (29.5 vs. 50.7%). Estimated average CVRFs by race across the life course are shown in Table 1. All CVRFs increased with age for both White and Black participants. In late-life, Black participants on average had higher values for all four CVRFs compared to White participants (Chi-Square test of independence, all *p*-values <0.001).

**Figure 1:**
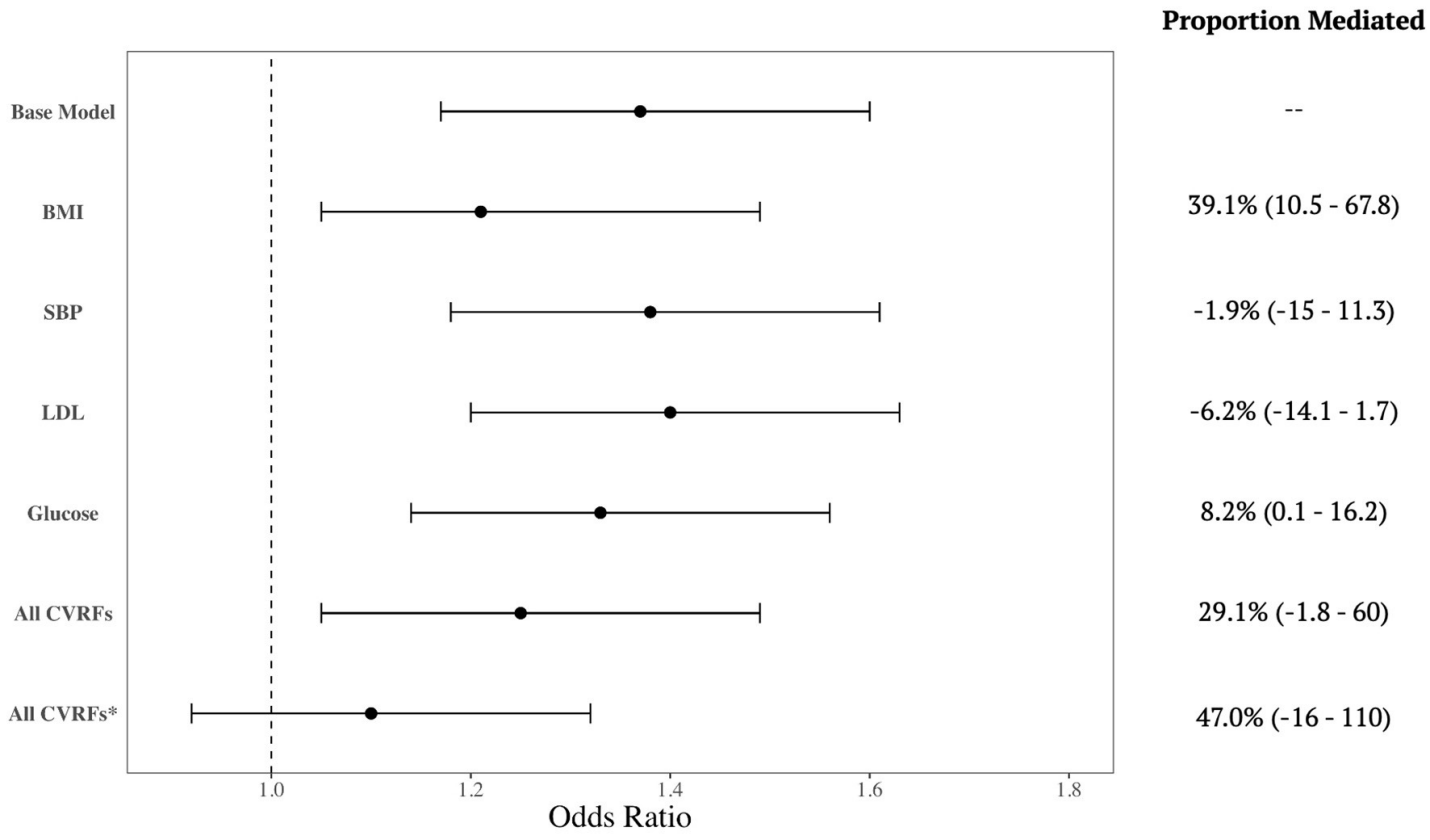
Mediation by cardiovascular risk factors of the relationship between race and dementia incidence. Forest plot shows the crude association and then additionally adjusted for each cardiovascular risk factor (CVRF), separately and all CVRFs together. Mediation effect is summarized by the proportion (%) of the association incidence that is mediated by the CVRF. All models are adjusted for age and sex, except for the last row (*) which is additionally adjusted for education.

There were 908 incident cases of dementia in the cohorts. Black participants had 1.37 (95% CI: 1.17–1.60) times the odds of developing dementia compared to White participants adjusted for age and sex. When included in the model individually, all CVRFs were statistically significant predictors of dementia. BMI was a notable mediator between race and dementia risk, accounting for 39.1% of the effect (95% CI: 10.5–67.8%). Glucose was also a mediator of this risk, accounting for 8.1% (95% CI: 0.1–16.2%) of the effect (Figure 1); however, SBP and LDL did not mediate the association. When all four CVRFs were included in a single model, they together mediated 29.1% of the association, although this was a trend-level significance (95% CI: −1.8–60%). When we additionally adjusted for education in these models, none of the individual CVRFs were statistically significant mediators. The joint effect of all CVRFs were no longer statistically significant predictors of dementia, but the confidence interval was imprecise, and the joint effect remained consistent with substantial mediation of the relationship between race and dementia risk (Proportion mediated: 47%; 95% CI: −16–110%).

In the CHS and Health ABC cohorts, 6,091 individuals had available cognitive scores. On average, Black participants scored 29.3 (14.1) on the DSST and 87.5 (8.65) on the 3MS, while White participants scored 40.2 (12.3) and 92.0 (5.15) respectively. The four CVRF risk factors were significant joint mediators of the effect of race on DSST score, explaining 12.0% of the association (95% CI: 8.5–15.5%, Table 2). Similar results were found for 3MS score, with the four CVRFs mediating 10% of the overall effect (95% CI: 6.2–13.8%, Table 2), mostly due to contributions by BMI and SBP. When additionally adjusting for education, the joint mediation effect of the CVRFs on DSST and 3MS scores were slightly attenuated but remained statistically significant.

**Table 2:** Mediation by cardiovascular risk factors on cognitive scores.

**Cardiovascular risk factor**	**DSST**	**3MS**
Body Mass Index	9.3% (6.5–12.1)	9.1% (5.8–12.4)
Systolic Blood Pressure	7.2% (5.3–9.0)	4.9% (3.0–6.7)
Low-density Lipoprotein Cholesterol	−1.1% (−2.0 to −0.2)	−1.7% (−2.6 to −0.7)
Glucose	1.0% (−0.1–2.2)	0.7% (−0.2–1.6)
All 4 CVRFs	12.0% (8.5–15.5)	10.0% (6.2–13.8)
All 4 CVRFs^*^	10.5% (6.9–14.2%)	8.3% (4.2–12.4)

Mediation, summarized as proportion mediated (%), of cardiovascular risk factors (CVRFs) between race and Digit Symbol Substitution Test (DSST) and Modified Mini-Mental State (3MS) scores. CVRFs are included in models as categorical cumulative period-specific TWAs. All models are adjusted for age and sex, except for the last row (^*^) which is additionally adjusted for education.

## 4. Discussion

Overall, we found that CVRFs contribute to racial disparities in dementia risk, especially BMI and fasting glucose, and the effect of all four CVRFs together accounted for almost a third of the variance, but this was of borderline statistical significance. All four CVRFs together were also mediators of processing speed and global cognition, which is further supportive of an overall mediative effect of CVRFs on dementia. After additional adjustment for education, another potential mediator, the joint contribution of all four CVRFs on cognitive scores remained significant.

The Baron and Kenny mediation framework makes two main assumptions: (1) there is no interaction between the exposure and mediator and (2) there is no unmeasured confounding of the mediator and outcome. First, we hypothesize that it is the difference in prevalence of CVRFs by race that leads to disparities in dementia incidence, rather than differing effects of CVRFs by race. There is prior evidence to support this hypothesis, with previous literature finding no interaction between CVRF and race on cognition (Yaffe et al., 2020; Peterson et al., 2021). Second, as is true with any observational study, there is the potential for unmeasured confounding between CVRFs and dementia. However, we have identified the most important confounders (age, sex, and education) and adjusted for those in our analysis. As such, we believe the assumptions made by this mediation analysis are reasonably met.

These results provide compelling evidence on modifiable risk factors which may contribute to racial disparities in dementia incidence. In this study, we utilized innovative methods to address restrictions on longitudinal data faced by previous studies with limited follow-up and no entire life course observations. Our results are consistent with another pooled cohort study that reported that cumulative lifetime SBP contributes to racial disparities in late-life cognition (Levine et al., 2020). Our conclusions are also supported by previous literature reporting that CVRFs are important risk factors for dementia (Whitmer et al., 2005; Alonso et al., 2009; Qiu and Fratiglioni, 2015; Livingston et al., 2020) and exposure differs by race (Lewey and Choudhry, 2014; Howard et al., 2017; Bell et al., 2018). Various mechanisms have been proposed for the differences in the prevalence of CVRFs between White and Black populations, such as racism (Cozier et al., 2006, 2014; Brondolo et al., 2011; Aaron and Stanford, 2021), chronic stress (Low et al., 2009), and access to timely and quality health care (Churchwell et al., 2020; Yearby et al., 2022). While the specific mechanisms for this pathway are beyond the scope of this report, it is important to recognize the contribution of systemic racism and social determinants of health (SDOH) to these disparities in CVRFs and dementia (Caunca et al., 2020; Zeki Al Hazzouri et al., 2022). Data limitations prevented this analysis from considering the contributions of important SDOH on dementia disparities, such as income and access to healthcare. However, our analysis revealed that education accounted for part of the contribution of CVRFs on dementia and cognition. Since education, and likely also socioeconomic status, play a crucial role in contributing to poor cognitive outcomes, more work is needed to understand the impact of SDOH on these relationships between CVRFs and dementia disparities.

Due to data limitations, we were unable to adjust for genetic factors such as *APOE*. The *APOE* ε*2* and ε*4* alleles are known to be more prevalent among Black individuals, but the association with cognition and AD is weaker among this group (Rajan et al., 2017, 2019; Beydoun et al., 2021). Since the *APOE* gene is also associated with increased of cardiovascular disease, this genetic risk factor may contribute to the observed mediating role of CVRFs (Mahley, 2016; Rajan et al., 2017). We hope in the future to conduct analyses with complete genetic data in a racially diverse sample to replicate the present analysis with adjustment for *APOE*.

Our study has important limitations. Most notably, this analysis was restricted to Black and White participants. Disparities in dementia incidence and treatment are documented among other racial groups such as Hispanic and Asian individuals (Mayeda et al., 2016; Chen and Zissimopoulos, 2018; Kawas et al., 2021). Future analyses would benefit from replicating this analysis in cohorts with representation of more diverse racial groups. Additionally, this sample had unbalanced sample sizes of Black and White individuals, meaning that we may have lacked sufficient power to detect all mediating effects of CVRFs. This analysis also uses pooled and imputed data instead of measured longitudinal data which relies on the critical assumption that early life CVRFs were properly and accurately imputed. While error cannot be ruled out, this method was validated using a simulation study and has been used in previous publications (Zeki Al Hazzouri et al., 2019; Brenowitz et al., 2021; Yaffe et al., 2021).

In summary, using novel imputation methods to model life course exposure to CVRFs, our results suggest that CVRFs contribute to racial disparities in dementia risk between Black and White individuals. This work contributes to previous research suggesting that CVRFs are key factors in dementia risk and subsequent disparities (Whitmer et al., 2005; Alonso et al., 2009; Qiu and Fratiglioni, 2015; Levine et al., 2020; Livingston et al., 2020; Yaffe et al., 2021; Zeki Al Hazzouri et al., 2021). Future studies should expand on CVRFs as important targets for reducing racial disparities in dementia and cognitive outcomes.

## Data availability statement

The data analyzed in this study is subject to the following licenses/restrictions: anonymized data from the cohort studies used in this analysis are available from each study's respective coordinating centers. Specific policies governing each study's data and the process to access data can be found online. Requests to access these datasets should be directed to CARDIA: cardia.dopm.uab.edu/; MESA: mesa-nhlbi.org/; CHS: www.chs-nhlbi.org/; Health ABC: healthabc.nia.nih.gov/.

## Ethics statement

The current analysis was approved by the Columbia University and University of California San Francisco IRBs approval. The patients/participants provided their written informed consent to participate in this study.

## Author contributions

KY, AZ, and EV were involved in conceptualizing and designing the study. KY, NA, and AF obtained the data. EV and EF conducted the statistical analysis. EF drafted the manuscript. All authors commented on subsequent versions and approved the submitted version.
